# The Trajectory Towards a Seasonally Ice-Free Arctic Ocean

**DOI:** 10.1007/s40641-018-0113-2

**Published:** 2018-09-26

**Authors:** Dirk Notz, Julienne Stroeve

**Affiliations:** 1 Max Planck Institute for Meteorology, Hamburg, Germany; 2 University College London, London, UK; 3 National Snow and Ice Data Center, Boulder, CO USA

**Keywords:** Sea ice, Arctic ocean, Future climate, Climate models, Global warming targets

## Abstract

**Purpose of Review:**

The observed substantial loss of Arctic sea ice has raised prospects of a seasonally ice-free Arctic Ocean within the foreseeable future. In this review, we summarize our current understanding of the most likely trajectory of the Arctic sea-ice cover towards this state.

**Recent Findings:**

The future trajectory of the Arctic sea-ice cover can be described through a deterministic component arising primarily from future greenhouse gas emissions, and a chaotic component arising from internal variability. The deterministic component is expected to cause a largely ice-free Arctic Ocean during summer for less than 2 ^∘^C global warming relative to pre-industrial levels. To keep chances below 5 % that the Arctic Ocean will largely be ice free in a given year, total future CO_2_ emissions must remain below 500 Gt.

**Summary:**

The Arctic Ocean will become ice free during summer before mid-century unless greenhouse gas emissions are rapidly reduced.

## Introduction

The observed recent loss of Arctic sea ice is probably the clearest indicator of the ongoing rapid changes in the climate state of our planet. Over the past few decades, the sea-ice-covered area of the Arctic Ocean has been reduced by about half during summer [1], and the ice thickness has also been reduced by about half [2]. In combination, this suggests that today’s volume of the Arctic summer sea-ice cover is only roughly a quarter of what it used to be just a few decades ago. In this review, we summarize our current understanding of how the remaining Arctic sea ice will evolve in the future.

We start with a brief overview of the main sources of information that have been used to estimate the future evolution of the Arctic sea-ice cover, focusing in particular on the strengths and weaknesses of the underlying models and observations. Based on this assessment, we afterwards provide a unified understanding of the response of the sea-ice cover to changes in external forcing, internal variability, and self amplification. This unified understanding is obtained by combining observation-based studies with model-based studies, allowing us to fully profit from the individual strengths of these two sources of information. In particular, we will demonstrate how quantitative estimates of the future evolution of the Arctic sea-ice cover can most robustly be obtained by combining observed quantitative information with robust underlying relationships obtained from model simulations.

## Sources of Information

As in most other scientific disciplines, we have two major sources of information to examine the loss of Arctic sea ice in the real world: observational estimates and models. As both these sources of information have certain limitations, the most robust insights into the ongoing and future evolution of Arctic sea ice are obtained from combining observations and models, ideally minimizing the impact of the limitations of either [3].

### Observations

Observational records of Arctic sea ice are the best possible estimate of the real evolution of the sea-ice cover. However, all existing observational records of Arctic sea ice have three distinct limitations, which imply that these observational records alone do not allow one to fully understand the drivers of the current ice changes or to fully characterize the future evolution of the sea-ice cover.

The first limitation is related to internal variability: Because the climate system is chaotic, any given set of external boundary conditions allows for an infinite number of possible climate trajectories. However, only one of these trajectories is actually realized and can be observed. This implies that observations can never capture the richness of possible trajectories that the climate system could have taken in recent decades. Hence, in the same way in which a single observation of the throwing of a dice only allows for a very limited understanding of the range of possible outcomes, a record of a climate observable often only allows for limited insights into the relative impact of external forcing or internal variability on the time evolution of that observable. In particular, all reliable observational records of sea ice only capture the behaviour of Arctic sea ice during a period of a rapidly changing background climate. Because of these rapid changes, even a 30-year long record is not long enough to provide a meaningful “average climate condition” that would sufficiently minimize the impact of internal variability [4, 5]. Their relatively short duration is hence a major drawback of all reliable records of sea-ice evolution.

The second limitation derives from observational uncertainty. In particular, all satellite records are based on indirect methods to obtain the underlying sea-ice property that one is ultimately interested in. Therefore, all major observational products are only an approximation of the true state of the sea-ice cover at any given time [6, 7].

The third limitation derives from the fact that we can only observe a very limited subset of climate variables that matter for the evolution of sea ice. For example, we have only limited observations of the major atmospheric heat fluxes that determine the surface energy balance of the ice cover, and no Arctic-wide observations of the oceanic heat flux underneath the ice.

If one keeps these limitations in mind, observational records can give us information about the sea-ice cover that offer a wealth of valuable insights into the past and also into the future evolution of Arctic sea ice. Indeed, we would argue that the most robust quantitative estimates of the future evolution of the ice cover are based on linear relationships between temperature or cumulative CO_2_ emissions and sea-ice coverage identified in models and observations, where the slope of the linear relationship is taken directly from the observational record.

The studies that we summarize here are primarily based on the 40-year long record of gridded Arctic sea-ice concentration as obtained from passive-microwave satellite observations. This record started in the late 1970s, and is based on a series of successive multi-frequency passive microwave sensors that allow scientists to observe the polar regions year-round regardless of polar night or cloud cover [8, 9]. The stark differences in emissivity between open water and ice allow for a binary classification of ice vs. no ice present. In reality, due to the coarse resolution, satellite pixels often contain a mixture of ice, open water, leads, etc., allowing for characterization of the fractional area of sea ice per satellite pixel. This is typically done through setting tie-points for open water and ice, and interpolating between these extremes to determine the sea-ice concentration. While current algorithms to convert the satellite observation to sea-ice cover are relatively robust during winter, once melt begins the sensitivity of the microwave emissivity to liquid water at the sea-ice surface can result in large underestimation of the true sea ice fraction within a satellite pixel [7]. Further, near the ice edge and within coastal regions, the large satellite footprint can result in false ice concentrations or underestimation of the actual ice edge location [10].

The studies that we discuss here primarily use the sea-ice area or the sea-ice extent derived from such satellite-retrievals of gridded sea-ice concentration. Arctic sea-ice area is usually calculated by multiplying sea-ice concentration with grid-cell area and adding up over all Northern-hemispheric grid cells. Arctic sea-ice extent is usually calculated by adding up the grid-cell area of all grid cells with at least 15% sea-ice coverage. In the following, we will use the term “sea-ice coverage” for any statement that is true for both sea-ice area and for sea-ice extent.

### Models

Any climate model has limitations that must be kept in mind when employing it to understand the evolution of the sea-ice cover in the real world.

First, climate models cannot capture all processes that govern the evolution of our climate system, and hence usually represent the real evolution of an observable less realistically than a given observational record. This is particularly true for sea ice, where climate-model simulations have been found to have substantial biases compared to the observed evolution of the ice cover. Among others, the models usually have a too low sensitivity of the simulated ice loss to simulated warming [3, 11–13] and to simulated CO_2_ emissions [14], they often have a too low or too high mean sea-ice area [15], they often have an erroneous distribution of ice thickness [16, 17], and they can have substantial biases in the albedo evolution throughout summer [18, 19]. These biases must be kept in mind when assessing the robustness of model-based studies on the future trajectory of Arctic sea ice.

Second, the relationship of a model to the real world is often very difficult to estimate [5], which makes it difficult to infer robust quantitative statements from model simulations. In particular, internal variability renders a perfect agreement of a model simulation with reality impossible, such that a disagreement between a model simulation and the real world does not allow one to directly obtain insights on the quality of a particular model simulation [5, 20]. Any model evaluation must hence take internal variability into account [15]. On the other hand, an agreement of a specific model simulation with some observed record does not necessarily indicate a reasonable description of the underlying processes in the numerical code of the model, but might instead just indicate a reasonable tuning of the model to match the observational record [5, 21] or be caused by compensating errors. For example, [22] suggest that models participating in the fifth phase of the Coupled Model Intercomparison Project (CMIP5 [23]) better reproduce the observed loss of Arctic sea ice compared to the models participating in CMIP3, because in CMIP5, the prescribed external forcing from volcanic eruptions is too strong. Furthermore, agreement of a model with the past evolution of an observable is not necessarily an indication for reliable projections of the future evolution of that observable [3, 5].

On the plus side, however, models overcome many of the mentioned limitations of observational records: models can be run several times and over long periods to capture the range of possible internal variability, they provide full three-dimensional fields of all major climate variables at nearly any desired temporal resolution, and they are internally consistent. In addition, they reflect possible future changes in the physical processes that drive the evolution of the sea-ice cover. Such future changes can obviously not be observed, which is why model simulations contribute important insights into the future evolution of the ice cover that can not be inferred from the observational record.

Most recent studies that employ climate-model simulations to explore the ongoing and future evolution of Arctic sea ice are based on the coordinated set of simulations from about 40 different climate models that participated in CMIP5. In addition, some recent studies are based on large ensembles of simulations with individual models. Such single-model large ensembles have been used for sea-ice-related studies for the Community Earth System Model (CESM [24]), the Canadian Earth System Model (Can-ESM [25]), and the Max Planck Institute Earth System Model (MPI-ESM). These large ensembles allow for robust insights into the impact of internal variability in individual models.

## The Impact of External Forcing

Changes in the external forcing of the sea-ice cover have been established as the major driver of the observed loss of Arctic sea ice in the vast majority of related recent studies [14, 22, 26, 27]. Internal variability, that we consider in more detail in the next section, has been found to have amplified the externally forced sea-ice loss, but the magnitude of the amplification is not clear yet [20, 28, 29].

### Past Evolution of the Sea-Ice Cover

The major role of changes in the external forcing for the past evolution of Arctic sea ice has been identified both in the observational record and in model simulations. In particular, both in models and in observations the Arctic sea-ice coverage (i.e., sea-ice area or sea-ice extent) during summer is linearly related to the rise in global-mean temperature [3, 11, 12, 22, 30–32]. [14] suggest a simple conceptual model to explain the linearity, establishing an underlying causal relationship between temperature rise and Arctic sea-ice loss. Linear correlations have also been established between the evolution of Arctic summer sea ice and atmospheric CO_2_ concentration [26, 33] and cumulative anthropogenic CO_2_ emissions [14, 34, 35]. These individual correlations are directly related to each other, because anthropogenic emissions of CO_2_ are also the main driver of the observed warming of the atmosphere [27], and the relationship between temperature rise and CO_2_ emissions has been largely linear in the past.

The linear relationships between the external drivers and Arctic sea-ice coverage do not only hold during summer, but have been shown to hold for all months, both for temperature [36] and for cumulative CO_2_ emissions [37]. For the linear regression against CO_2_, *R*^2^ values range between 0.75 and 0.92 for every month of the year over the period 1953–2017 [37]. This suggests that the majority of the ice loss across all seasons can directly be explained by the anthropogenic release of CO_2_.

Other changes in external forcing have only had a limited impact on the sea-ice cover in the past few decades. For example, the observational record shows no substantial co-variation of Arctic sea ice with changes in solar activity [26]. In particular, the slight weakening of solar activity since the early 2000s is inconsistent with the ongoing rapid loss of Arctic sea ice. Large observed volcanic eruptions such as El Chichon in 1982 and Mount Pinatubo in 1991 caused a small increase in simulated sea-ice coverage in the ensemble mean of large ensemble simulations, but their impact can not be identified in individual simulations [29]. This is particularly relevant given that the impact of volcanic eruptions has been suggested to be overestimated in CMIP5 model simulations [13]. Hence, natural changes in the external forcing have not played a major role for the observed evolution of the Arctic sea-ice cover.

Also anthropogenic aerosols have probably been of limited importance for the observed evolution of the Arctic sea-ice cover in recent decades. This is because their abundance has remained largely unchanged in the recent past, which is why it is unlikely that aerosols contributed much to the observed rapid loss of sea-ice evolution [38].

### Future Evolution of the Sea-Ice Cover

The sensitivity of Arctic sea ice as described by the linear relationship between global-mean temperature and Arctic sea-ice coverage has been found to remain constant in model simulations across a wide spectrum of temperature trajectories [31, 32, 39]. In particular, the linearity holds in all CMIP5 models until summer sea ice vanishes in individual simulations. Hence, the observed sensitivity can be extrapolated to directly estimate the response of the Arctic sea-ice cover to future warming.

Unfortunately, as shown by the values for the September sea-ice cover listed in Table 1, uncertainty exists as to the true sensitivity of the sea-ice cover to a given amount of warming. The main sources of uncertainty are the choice of the underlying observational records of sea-ice coverage and global-mean temperature, and the method used to estimate the sensitivity from any given pair of observational records. Regarding the latter, [13] divide the temporal trend of sea-ice coverage by the temporal trend of global-mean temperature to estimate sea-ice sensitivity, while all other estimates in Table 1 are based on an ordinary least-square regression of sea-ice coverage on global-mean temperature. This latter approach has been suggested to underestimate the true sensitivity of the sea-ice cover [11], as it implies a primarily unidirectional relationship between the two variables. Therefore, the inverse of temperature regressed on sea-ice area gives a higher estimate than the direct regression of sea-ice area on temperature. However, one could also argue that the simple linear regression is justified by the primarily one-way causal relationship between global warming and sea-ice loss, suggesting that this estimate reflects the true sensitivity.

**Table 1:** Overview of published sensitivities of the September sea-ice cover to global warming

Sensitivity	Ice-free at	Sea-ice data	Temperature data	Time period	Ref
[10^6^ km^2^/K]					
Based on sea-ice extent					
− 5.7^+^	+ 0.6 ^∘^C	NSIDC sea-ice index	GISTEMP	1979–2013	[13]
− 4.4	+ 0.8 ^∘^C	NSIDC sea-ice index	GISTEMP	1979–2014	[40]
Based on sea-ice area					
− 2.6	+ 0.9 ^∘^C	HadISST 1^∗^	GISTEMP	1979–2007	[12]
− 4.1	+ 0.6 ^∘^C	Sea-ice index & HadISST 1^∗^	HadCRUT4	1953–2016	[36]
− 3.3	+ 0.7 ^∘^C	HadISST 2.2	GISTEMP	1953–2016	[36]

The first column is the estimated observed sea-ice loss per degree of global warming. The second column is the additional warming above present levels needed to obtain an Arctic sea-ice coverage of less than 1 million km^2^ (based on average September sea-ice extent of past ten years of 4.7 million km^2^ and average September sea-ice area of 3.3 million km^2^ [1]). ^∗^Note that [41] identified an inconsistency in the HadISST 1 sea-ice area, and recommended that it should only be used when merged with a consistent satellite record. ^+^This estimate is based on the gradient ratio of the temporal trend of global-mean warming and of the temporal trend of sea-ice coverage. All other estimates are based on ordinary regression of sea-ice coverage on global-mean temperature (see [11] for details)

Despite these uncertainties, the different estimates result in a relatively narrow range of additional warming above present that is required to obtain a near-ice free Arctic Ocean during summer, defined as the total sea-ice coverage dropping below 1 million km^2^. Based on an average sea-ice extent during September of 4.7 million km^2^ and an average sea-ice area during September of 3.3 million km^2^ as given by the NSIDC sea-ice index for the past 10 years [1], the additional warming needed to reduce the sea-ice coverage to less than 1 million km^2^ is found to be between + 0.6 and + 0.9 ^∘^C relative to the average global-mean temperature of the past ten years (Table 1). As current warming levels are about + 1 ^∘^C above pre-industrial levels, the Arctic Ocean can be expected to have an average ice coverage of less than 1 million km^2^ during summer for less than 2 ^∘^C global warming.

Estimates from model simulations that either capture the observed sensitivity or have been bias-corrected, result in similar estimates [36, 39, 42, 43]. Because of the robust relationship between sea-ice coverage and global-mean temperature, the time scale for establishing a given warming is irrelevant. As soon as the global-mean temperature has risen by slightly below 2 ^∘^C, the Arctic Ocean is expected to be on average nearly ice-free during September.

**Fig. 1:**
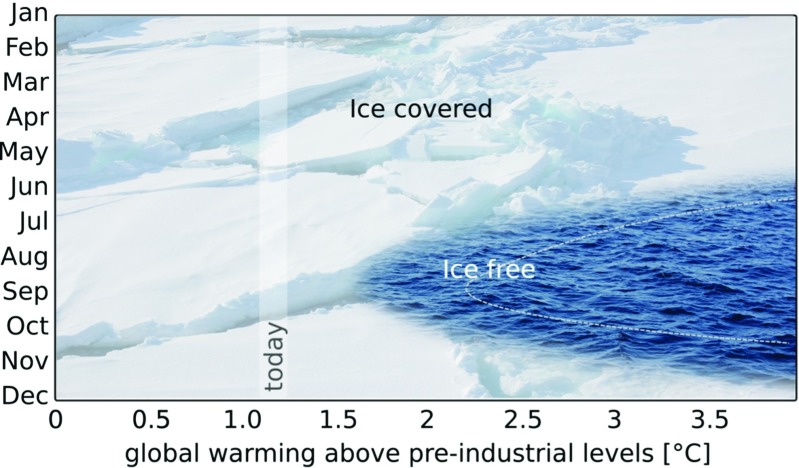
Estimated evolution of the Arctic sea-ice cover in response to mean global warming. The figure is based on the extrapolation of the linear relationship between monthly mean Arctic sea-ice area and global-mean temperature provided by [36]. The ice-free range of their high-sensitivity observational record is shown as the water-filled area, while the ice-free range of their low-sensitivity observational record is shown as the dashed line. The estimated warming until 2018 is marked with “today”

The linearity can also be exploited to estimate the future seasonal cycle of the Arctic sea-ice cover directly from the observational record (Fig. 1). This is because the linearity holds for every month of the year [36].

With temperature being the main driver of the long-term sea-ice evolution of the Arctic, regardless of the drivers for that temperature increase, the future evolution of the sea-ice cover is most robustly described in reference to this variable. However, because the observed near linear relationship between global-mean temperature and cumulative anthropogenic CO_2_ emissions has been found to very likely remain unchanged for the foreseeable future [27, 44–47], this relationship can be exploited to describe the future evolution of the sea-ice cover in response to cumulative future CO_2_ emissions [14]. The observed sensitivity of the Arctic sea-ice cover to cumulative anthropogenic CO_2_ emissions suggests an on average ice-free Arctic Ocean throughout August and September for an additional maximum 800 Gt of anthropogenic CO_2_ emissions. This estimate is based on the observed sensitivity of about 3-m^2^ sea-ice-area loss per ton of CO_2_ emissions in these months [37] and the average sea-ice area of the past 10 years. For an additional 1400 Gt of anthropogenic CO_2_ emissions, the Arctic Ocean will become largely sea-ice free from July to October [37].

A possible modification of these estimates might be caused by the future evolution of anthropogenic aerosols, as they are expected to become less abundant over the next few decades. While in the past decades, rather constant levels of anthropogenic aerosols have minimized the impact of aerosols on the loss of Arctic sea ice [38], in climate-model simulations the expected aerosol reduction causes additional ice loss relative to the one driven by anthropogenic CO_2_ emissions [38, 48]. This would imply that the estimates given here are too conservative.

A substantial number of studies that have examined the possible occurrence of an ice-free Arctic Ocean have not estimated the necessary warming, but instead describe the future evolution of the sea-ice cover primarily as a function of time [17, 27, 49–51]. However, time is not a control variable of the system. Instead, the link to time is established via the global-mean warming in a given year, which in turn is primarily a function of cumulative future emissions. Hence, any estimate of a given year during which the Arctic will lose its ice cover will always be as uncertain as the future evolution of anthropogenic CO_2_ emissions, which makes it impossible to estimate the future evolution of the Arctic sea-ice cover as a function of time alone.

## The Impact of Internal Variability

While the observed linear relationship between sea-ice coverage and global-mean temperature allows one to estimate the long-term average future evolution of the pan-Arctic ice cover, the evolution of the real ice cover will show substantial year-to-year variability because of internal variability. This year-to-year variability must be considered if one examines the possible upper and lower bounds of total sea-ice coverage for a specific amount of global warming. Our understanding of this internal variability of the sea-ice cover has substantially increased over the past few years, in particular because of the availability of large model ensembles which greatly ease the analysis of internal variability.

### Past Evolution of the Sea-Ice Cover

The observed evolution of the Arctic sea-ice cover is well described by the linear combination of an externally forced trend and superimposed internal variability [26]. However, estimating the true impact of internal variability in particular on the rapid ice loss in recent years is challenging, as the external forcing is changing rapidly. Hence, it is difficult to estimate the true internal variability of the sea-ice cover directly from the observational record. The pre-satellite record of sea-ice coverage from 1953 to 1979 as summarized in the HadISST record is assumed to be rather reliable [41] and does not show a strong trend. It provides a standard deviation of year-to-year changes in September sea-ice area from internal variability of *σ* = 0.36 million km^2^ [26].

CMIP5 models display on average a similar variability. In their pre-industrial control simulations, the average standard deviation of September sea-ice area is *σ* = 0.43 ± 0.12 million km^2^ [15]. Hence, based on a 2*σ* 95% confidence interval, the models and the observations suggest a maximum impact of internal variability on September sea-ice coverage of less than ± 1 million km^2^ for any given year for pre-industrial ice coverage. The internal variability depends on the sea-ice-covered area and reaches in model simulations a maximum at a sea-ice extent of around 3 million km^2^ [25]. With a further decrease in sea-ice cover, internal variability decreases towards pre-industrial values and eventually drops to zero as ice-coverage drops to zero [25]. As in the following, we are primarily interested in internal variability for low ice coverage, we take the pre-industrial value as a conservative estimate for the relevant internal variability of a future low ice state. This is in line with the finding that the long-term mean variability for the decreasing ice coverage of a high-emission scenario is across the CMIP5 models very similar to the pre-industrial long-term mean variability [15].

Examining the observed and modeled trends, a number of studies have found that a range of internal variability of ± 1 million km^2^ is sufficient to explain much of the biases in modeled sea-ice trends over the past few decades [5, 15, 20, 29]. Given that most models underestimate the observed sea-ice loss, this finding suggests that the observed ice loss has recently been amplified by internal variability [20, 29]. This is in line with a more process-oriented study examining the link between atmospheric variability and the resulting evolution of the sea-ice cover [28]. This might also explain why sea-ice loss has slowed down during summer since 2012, as the ice cover is ”recovering” from an extreme-event ice loss to the long-term trend line provided by the external forcing. A possible explanation for this behavior relates to changes in ocean circulation arising from internal variability [52].

### Future Evolution of the Sea-Ice Cover

Internal variability will remain superimposed on any externally forced trend. Hence, for a given year, the expected ice coverage can be calculated as the expected externally forced ice coverage based on the mean global air temperature plus/minus a specific ice coverage given by internal variability. Given the estimated 95% likelihood interval of at maximum ± 1 million km^2^ in sea coverage, we can use the observed sensitivity to translate the internal variability of sea-ice coverage to likelihood ranges for any given linear driver of sea-ice evolution. By dividing 1 million km^2^ through the observed sensitivities summarized in Table 1, we find that the temperature at which the Arctic first becomes ice free will have a 95% uncertainty range from internal variability of ± 0.2 to 0.4 ^∘^C. These values are in line with results from large-ensemble model simulations [36]. Combined with the temperature threshold of less than + 2 ^∘^C for a near ice-free ocean during summer, this then implies low, but above zero chances for a near-ice free Arctic ocean at + 1.5 ^∘^C global warming. Again, this is in line with existing recent studies [36, 39, 42, 43].

The observed relationship between Arctic sea-ice loss and cumulative CO_2_ emissions allows us to translate the observed internal variability of the sea-ice cover to an uncertainty range of cumulative CO_2_ emissions at which Arctic sea ice will be lost during summer. Dividing the 95% likelihood interval of Arctic sea-ice area of ± 1 million km^2^ by the observed sensitivity of − 3 million km^2^/(1000 Gt) directly gives an uncertainty range of ± 330 Gt of cumulative CO_2_ emissions for the estimate of the occurrence of an ice free Arctic Ocean during summer. Hence, the 95% uncertainty range for cumulative future emissions of CO_2_ leading to an ice free Arctic Ocean during summer becomes 500 to 1100 Gt based on the average value of 800 Gt established above. For a given emission per year, this uncertainty range can be translated to an uncertainty range of the year when the Arctic first becomes ice free. For today’s emission of about 40 Gt CO_2_ per year, we get an uncertainty range of about 15 years, similar to the range of 20 years estimated in earlier studies based on model simulations [5, 51].

## The Impact of Self-Amplification

In addition to changes in the external forcing and internal variability, also a possible self-amplification of the ice loss has been suggested to contribute to the substantial ice loss in recent years. This has often been framed in the context of nonlinear threshold behavior of the ice cover, also referred to as “tipping points.” The main mechanism that has been suggested to possibly give rise to such “tipping” of the ice cover has been the ice-albedo feedback.

### Past Evolution of the Sea-Ice Cover

The observed evolution of the Arctic sea-ice cover is inconsistent with the possible existence of a tipping point arising from self-amplification of the ice cover. First, the evolution has remained linearly linked to the long-term rise in global-mean temperature as outlined above. Second, the 1-year lag autocorrelation of year-to-year changes in summer sea-ice coverage is negative (around − 0.5, see also [26]), implying that after a summer with a particularly strong ice loss, the ice cover usually recovers somewhat in the following year. If indeed self-amplification played a significant role in the observed evolution of the ice cover, one would expect both an increasing failure of the linear relationship with rising temperature and an even more substantial ice loss after a year with a substantial ice loss.

Several stabilizing feedbacks during winter contribute to the lack of self-amplification even in light of the ice-albedo feedback [53]: First, any ice-free parts of the Arctic Ocean more effectively lose their heat to the atmosphere than those parts that remained ice-covered during winter [54]. Second, thin ice grows much faster than thick ice in response to the same external forcing, allowing for some recovery of total sea-ice volume after any record ice loss during summer [55]. And third, the later the ice cover forms, the thinner will be its isolating snow cover during winter [56].

Note that all these stabilizing feedbacks are only active during winter. Throughout summer of a given year, any small perturbation of the ice cover will be effectively amplified. For example, the observed progressively earlier melt onset translates directly to more absorption of solar heat, and thus to a more substantial ice loss during summer and a later onset of autumn freeze-up. This behavior has been found in the observational record [57] and can be used to estimate the timing of freeze onset at the end of the summer [58].

The observed lack of self-amplification of the ongoing ice loss is consistent with the behaviour of the Arctic sea-ice cover in large-scale climate models. In contrast, simplified models of the sea-ice cover often show instabilities [59]. This behavior has been explained by the lack of meridional heat transport and a lack of a seasonal cycle in these simplified models [60].

### Future Evolution of the Sea-Ice Cover

Also for the future, no substantial self amplification of the summer ice loss is expected. In particular, in all models that participated in CMIP5, the linear relationship between Arctic sea-ice cover and global-mean temperature holds until all sea ice is lost. This behavior already takes into account that in many regions, the ice-free duration during summer is becoming longer and longer, and that the ice cover as a whole is getting thinner. These factors are apparently not sufficient to overcome the stabilizing feedbacks and do not cause an acceleration of the summer sea-ice loss.

In contrast, the pace of winter sea-ice loss accelerates once the total area covered in summer approaches nearly ice-free conditions, both in idealized studies [61] and in many CMIP5 models [62, 63]. This behavior has recently been explained by geometric reasons, since the winter sea-ice covering the Arctic Ocean after the loss of summer sea ice will have a rather small homogeneous thickness and can thus be removed quickly [63, 64].

## Open Questions

As summarized in the past sections, over the last few years, we have gained substantial understanding of the past and future evolution of the ice cover. The most robust of these insights have been derived from a combination of model simulations and observational records, primarily records of sea-ice concentration. Based on this recent progress, we believe that we have a good first-order understanding of the trajectory of the future Arctic Ocean towards a possibly seasonal ice cover. However, many of the details of this trajectory remain poorly understood. We see the following four issues as the most important open questions regarding our understanding of the past and future trajectory of the Arctic sea-ice cover.

First, we lack understanding of the different pathways by which the ocean and the atmosphere translate changes in the external forcing and their own internal variability into changes of the ice cover. For example, the pathways by which heat is transported to the central Arctic Ocean are not clear. A recent study found that in 11 CMIP5 models, the heat required to melt the ice cover enters the Arctic via the atmosphere. In 11 other CMIP5 models, it enters the Arctic via the ocean, and in four CMIP5 models, both oceanic and atmospheric pathways supply net heat to the Arctic [65]. This uncertainty is also reflected by the split between either atmospheric [28, 66, 67] or oceanic [52, 68, 69] processes that have been suggested to be the main contributor to the observed evolution of the Arctic ice cover.

Second, we lack understanding of the regional evolution of the ice cover. For example, a regional analysis of trends in Arctic sea-ice coverage over recent decades has established substantial differences in response to the large-scale global warming [37, 70]. To understand these regional differences, we need to understand how the large-scale changes in the forcing are regionally translated into changes in the dynamics and thermodynamics of the ice cover. So far, only few studies address this topic. For example, variability of sea ice in the Barents Sea has been linked to large-scale changes in atmospheric flow regimes as captured by the North-Atlantic-Oscillation (NAO, [71, 72]), raising the possibility of a slow down of regional sea-ice loss in this region with a shift in the NAO index.

Third, we lack understanding of many of the implications of the ongoing ice loss. For example, a number of studies have suggested that the large-scale sea-ice loss might affect atmospheric circulation patterns and mid-latitude weather systems (see, for example, reviews [73–76]). In observational records, such link is probably hidden among substantial internal variability [76]. In modeling studies, the importance of a possible link depends critically on the experimental design [77], raising questions on the robustness of inferred linkages in existing studies.

Fourth, we lack a good understanding of the past and future evolution of sea-ice volume. This is related to the lack of a sufficiently long and sufficiently reliable observational record of sea-ice volume, to uncertainties in reanalyzed sea-ice volume [78], and to a failure of many CMIP5 models to reproduce the observed distribution and time evolution of sea-ice thickness [16, 17].

## Conclusion

In this review, we have demonstrated the consistency of existing observation-based studies with existing modeling-based studies regarding the future evolution of sea ice. In particular, we have demonstrated how we can use observational records to reproduce many recent quantitative estimates from primarily model-based studies. The quantitative link between models and observations is established as the agreement with the observed sensitivity has become a litmus test for any model-based estimate of the future evolution of the Arctic sea-ice cover. Where the observed sensitivity is not reproduced by a model, bias correction is applied to obtain reliable model-based estimates of the future evolution of the sea-ice cover. This also implies that often, the best estimate of the future evolution of the Arctic sea-ice cover is obtained by establishing robust relationships in models and observations, and to then extrapolate the quantitative manifestation of such robust relationships from the observational record into the future. The major uncertainty then derives from the quantification of the true sensitivity of the ice cover to changes in the external forcing.

Based on this approach, we identify the following statements to be very likely true for the future evolution of the Arctic sea-ice cover:

1. The Arctic sea-ice cover has been and will remain linearly related to global-mean air temperature in all months. Global-mean air temperature can hence be interpreted as the most important control variable on future Arctic sea-ice evolution.
2. The observed linear relationship between Arctic sea-ice coverage and global-mean air temperature suggests Arctic sea-ice coverage to drop below 1 million km^2^ in more than 50% of all years for a global warming of less than 2 ^∘^C compared to pre-industrial levels.
3. The observed linear relationship between Arctic sea-ice coverage and cumulative anthropogenic emissions of CO_2_ suggests Arctic sea-ice coverage to below 1 million km^2^ for more than 50% of all years for total future anthropogenic CO_2_ emissions of less than 800 Gt.
4. From internal variability, September sea-ice coverage can vary by a maximum of ± 1 million km^2^ for a given global-mean air temperature.
5. This year-to-year fluctuation can directly be translated into an uncertainty of ± 0.2 to 0.4 ^∘^C for the global warming at which the Arctic Ocean loses its summer sea ice for the first time. For CO_2_ emissions, the uncertainty is about ± 300 Gt CO_2_ emissions. Hence, the Arctic Ocean can be expected to be nearly ice-free in 5% of all years for 500-Gt future CO_2_ emissions and in 95% of all years for 1100-Gt future CO_2_ emissions.
6. As the observed linear relationship between Arctic sea-ice coverage and global-mean temperature currently hold for all months, they allow us to estimate the future seasonal cycle directly from the observational record. This is also true for the observed linear relationship between Arctic sea-ice coverage and cumulative CO_2_ emissions.

Based on current emission rates of about 40-Gt CO_2_ per year, these findings imply a substantial likelihood of an ice-free Arctic Ocean during summer before mid-century. The time window to prevent the loss of the Arctic summer sea-ice cover hence closes very rapidly.

## References

[1] Fetterer F, Knowles K, Meier W, Savoie M. 2018. Sea ice index: National Snow and Ice Data Center, Boulder. 2002 updated.

[2] Kwok R, Rothrock DA (2009). Decline in Arctic sea ice thickness from submarine and ICESat records: 1958–2008. Geophys Res Lett.

[3] Stroeve J, Notz D (2015). Insights on past and future sea-ice evolution from combining observations and models. Glob Planet Change.

[4] Arguez A, Vose RS (2010). The definition of the standard WMO climate normal: the key to deriving alternative climate normals. Bull Amer Meteor Soc.

[5] Notz D (2015). How well must climate models agree with observations?. Phil Trans R Soc A.

[6] Notz D (2014). Sea-ice extent and its trend provide limited metrics of model performance. Cryosphere.

[7] Ivanova N, Pedersen LT, Tonboe RT, Kern S, Heygster G, Lavergne T (2015). Inter-comparison and evaluation of sea ice algorithms: towards further identification of challenges and optimal approach using passive microwave observations. Cryosphere.

[8] Cavalieri DJ, Parkinson CL, Gloersen P, Zwally HJ. Arctic and antarctic sea ice concentrations from multichannel passive-microwave satellite data sets: October 1978 to December 1996. NASA; 1997. 104647.

[9] Comiso JC, Cavalieri DJ, Parkinson CL, Gloersen P (1997). Passive microwave algorithms for sea ice concentration. A comparison of two techniques. Remote Sens Env.

[10] Meier WN (2005). Comparison of passive microwave ice concentration algorithm retrievals with AVHRR imagery, in Arctic peripheral seas. IEEE Trans Geosci Remote Sens.

[11] Winton M (2011). Do climate models underestimate the sensitivity of Northern hemisphere sea ice cover?. J Clim.

[12] Mahlstein I, Knutti R (2012). September Arctic sea ice predicted to disappear near 2 ∘C global warming above present. J Geophys Res.

[13] Rosenblum E, Eisenman I (2017). Sea ice trends in climate models only accurate in runs with biased global warming. J Clim.

[14] Notz Dirk, Stroeve Julienne (2016). Observed Arctic sea-ice loss directly follows anthropogenic CO 2 emission. Science.

[15] Olonscheck D, Notz D (2017). Consistently estimating internal climate variability from climate model simulations. J Clim.

[16] Stroeve J, Barrett A, Serreze M, Schweiger A (2014). Using records from submarine, aircraft and satellites to evaluate climate model simulations of Arctic sea ice thickness. Cryosphere.

[17] Melia N, Haines K, Hawkins E (2015). Improved Arctic sea ice thickness projections using bias-corrected CMIP5 simulations. Cryosphere.

[18] Karlsson J, Svensson G (2013). Consequences of poor representation of Arctic sea-ice albedo and cloud-radiation interactions in the CMIP5 model ensemble. Geophys Res Lett.

[19] Koenigk T, Devasthale A, Karlsson KG (2014). Summer Arctic sea ice albedo in CMIP5 models. Atmos Chem Phys.

[20] Swart NC, Fyfe JC, Hawkins E, Kay JE, Jahn A (2015). Influence of internal variability on Arctic sea-ice trends. Nat Clim Change.

[21] Mauritsen T, Stevens B, Roeckner E, Crueger T, Esch M, Giorgetta M (2012). Tuning the climate of a global model. J Adv Model Earth Syst.

[22] Rosenblum E, Eisenman I (2016). Faster Arctic sea ice retreat in CMIP5 than in CMIP3 due to volcanoes. J Climate.

[23] Taylor KE, Stouffer RJ, Meehl GA (2012). An overview of CMIP5 and the experiment design. Bull Amer Meteor Soc.

[24] Kay JE, Deser C, Phillips A, Mai A, Hannay C, Strand G (2014). The community earth system model (CESM) large ensemble project: a community resource for studying climate change in the presence of internal climate variability. Bull Amer Meteor Soc.

[25] Kirchmeier-Young MC, Zwiers FW, Gillett NP (2016). Attribution of extreme events in Arctic sea ice extent. J Clim.

[26] Notz D, Marotzke J (2012). Observations reveal external driver for Arctic sea-ice retreat. Geophys Res Lett.

[27] Stocker T F, Qin D, Plattner G K, Tignor M, Allen S K, Boschung J, et al., (eds). 2013. Climate change 2013: The physical science basis. Contribution of working Group I to the 5th assessment report of the intergovernmental panel on climate change. Cambridge: Cambridge University Press.

[28] Ding Q, Schweiger A, L’Heureux M, Battisti DS, Po-Chedley S, Johnson NC (2017). Influence of high-latitude atmospheric circulation changes on summertime Arctic sea ice. Nat Clim Change.

[29] Notz D (2017). Arctic sea ice seasonal-to-decadal variability and long-term change. Past Global Changes Magazine.

[30] Gregory JM, Stott PA, Cresswell DJ, Rayner NA, Gordon C, Sexton DMH (2175). Recent and future changes in Arctic sea ice simulated by the HadCM3 AOGCM. GeophysResLett.

[31] Ridley JK, Lowe JA, Hewitt HT (2012). How reversible is sea ice loss?. Cryosphere.

[32] Li C, Notz D, Tietsche S, Marotzke J (2013). The transient versus the equilibrium response of sea ice to global warming. J Climate.

[33] Johannessen OM (2008). Decreasing Arctic sea ice mirrors increasing CO2 on decadal time scale. Atmospheric and Oceanic Science Letters.

[34] Zickfeld K, Arora VK, Gillett NP (2012). Is the climate response to CO2 emissions path dependent?. Geophys Res Lett.

[35] Herrington T, Zickfeld K (2014). Path independence of climate and carbon cycle response over a broad range of cumulative carbon emissions. Earth Syst Dynam.

[36] Niederdrenk AL, Notz D (2018). Arctic sea ice in a 1.5∘C warmer world. Geophys Res Lett.

[37] Stroeve Julienne, Notz Dirk (2018). Changing state of Arctic sea ice across all seasons. Environmental Research Letters.

[38] Wang Y, Jiang JH, Su H, Choi YS, Huang L, Guo J (2017). Elucidating the role of anthropogenic aerosols in Arctic sea ice variations. J Clim.

[39] Jahn Alexandra (2018). Reduced probability of ice-free summers for 1.5 °C compared to 2 °C warming. Nature Climate Change.

[40] Stroeve J, Notz D (2016). Corrigendum to insights on past and future sea-ice evolution from combining observations and models [Glob. Planet. Change (2015) 119–132]. Global Planet Change.

[41] Meier WN, Stroeve J, Fetterer F (2007). Whither Arctic sea ice? A clear signal of decline regionally, seasonally and extending beyond the satellite record. Ann Glaciol.

[42] Screen JA, Williamson D (2017). Ice-free Arctic at 1.5 ∘C?. Nat Clim Change.

[43] Sigmond Michael, Fyfe John C., Swart Neil C. (2018). Ice-free Arctic projections under the Paris Agreement. Nature Climate Change.

[44] Matthews HD, Caldeira K (2008). Stabilizing climate requires near-zero emissions. Geophys Res Lett.

[45] Matthews HD, Solomon S (2013). Irreversible does not mean unavoidable. Science.

[46] MacDougall AH, Friedlingstein P (2015). The origin and limits of the near proportionality between climate warming and cumulative CO2 emissions. J Clim.

[47] MacDougall AH (2016). The transient response to cumulative CO2 emissions: a review. Current Climate Change Reports.

[48] Gagné ME, Gillett NP, Fyfe JC (2015). Impact of aerosol emission controls on future Arctic sea ice cover. Geophys Res Lett.

[49] Massonnet F, Fichefet T, Goosse H, Bitz CM, Philippon-Berthier G, Holland MM (2012). Constraining projections of summer Arctic sea ice. Cryosphere.

[50] Wang M, Overland JE (2012). A sea ice free summer Arctic within 30 years: an update from CMIP5 models. Geophys Res Lett.

[51] Jahn A, Kay JE, Holland MM, Hall DM (2016). How predictable is the timing of a summer ice-free Arctic?. Geophys Res Lett.

[52] Zhang R (2015). Mechanisms for low-frequency variability of summer Arctic sea ice extent. Proc Natl Acad Sci.

[53] Notz D (2009). The future of ice sheets and sea ice: between reversible retreat and unstoppable loss. ProcNatlAcadSci.

[54] Tietsche S, Notz D, Jungclaus JH, Marotzke J (2011). Recovery mechanisms of Arctic summer sea ice. Geophys Res Lett.

[55] Bitz C, Roe G (2004). A mechanism for the high rate of sea ice thinning in the Arctic Ocean. J Clim.

[56] Hezel P. J., Zhang X., Bitz C. M., Kelly B. P., Massonnet F. (2012). Projected decline in spring snow depth on Arctic sea ice caused by progressively later autumn open ocean freeze-up this century. Geophysical Research Letters.

[57] Stroeve JC, Markus T, Boisvert L, Miller J, Barrett A (2014). Changes in Arctic melt season and implications for sea ice loss. Geophys Res Lett.

[58] Stroeve JC, Crawford AD, Stammerjohn S (2016). Using timing of ice retreat to predict timing of fall freeze-up in the Arctic. Geophys Res Lett.

[59] North GR (1984). The small ice cap instability in diffusive climate models. J Atmos Sci.

[60] Wagner TJW, Eisenman I (2015). How climate model complexity influences sea ice stability. J Clim.

[61] Eisenman I, Wettlaufer JS (2009). Nonlinear threshold behavior during the loss of Arctic sea ice. Proc Natl Acad Sci USA.

[62] Eisenman I, Schneider T, Battisti DS, Bitz CM (2011). Consistent changes in the sea ice seasonal cycle in response to global warming. J Clim.

[63] Drijfhout S, Bathiany S, Beaulieu C, Brovkin V, Claussen M, Huntingford C (2015). Catalogue of abrupt shifts in intergovernmental panel on climate change climate models. Proc Natl Acad Sci.

[64] Bathiany S, Notz D, Mauritsen T, Raedel G, Brovkin V (2016). On the potential for abrupt Arctic winter sea ice loss. J Climate.

[65] Burgard C, Notz D (2017). Drivers of Arctic Ocean warming in CMIP5 models. Geophys Res Lett.

[66] Kapsch ML, Graversen RG, Tjernström M (2013). Springtime atmospheric energy transport and the control of Arctic summer sea-ice extent. Nat Clim Change.

[67] Letterly A, Key J, Liu Y (2016). The influence of winter cloud on summer sea ice in the Arctic, 1983–2013. J Geophys Res Atm.

[68] Ȧrthun M, Eldevik T, Smedsrud LH, Skagseth Ø, Ingvaldsen RB (2012). Quantifying the influence of Atlantic heat on Barents Sea ice variability and retreat. J Clim.

[69] Miles MW, Divine DV, Furevik T, Jansen E, Moros M, Ogilvie AEJ (2013). A signal of persistent Atlantic multidecadal variability in Arctic sea ice. Geophys Res Lett.

[70] Onarheim Ingrid H., Eldevik Tor, Smedsrud Lars H., Stroeve Julienne C. (2018). Seasonal and Regional Manifestation of Arctic Sea Ice Loss. Journal of Climate.

[71] Luo B, Luo D, Wu L, Zhong L, Simmonds I (2017). Atmospheric circulation patterns which promote winter Arctic sea ice decline. Environ Res Lett.

[72] Lien VS, Schlichtholz P, Skagseth Ø, Vikebø FB (2017). Wind-driven Atlantic water flow as a direct mode for reduced Barents Sea ice cover. J Clim.

[73] Budikova D (2009). Role of Arctic sea ice in global atmospheric circulation: a review. Global Planet Change.

[74] Bader J, Mesquita MDS, Hodges KI, Keenlyside N, Østerhus S, Miles M (2011). A review on Northern Hemisphere sea-ice, storminess and the North Atlantic Oscillation: observations and projected changes. Atmos Res.

[75] Vihma T (2014). Effects of Arctic sea ice decline on weather and climate: a review. Surv Geophys.

[76] Barnes EA, Screen JA (2015). The impact of Arctic warming on the midlatitude jet-stream: Can it? Has it? Will it?. WIRES Clim Change.

[77] Screen JA, Deser C, Smith DM, Zhang X, Blackport R, Kushner PJ (2018). Consistency and discrepancy in the atmospheric response to Arctic sea-ice loss across climate models. Nat Geosci.

[78] Chevallier M, Smith GC, Dupont F, Lemieux JF, Forget G, Fujii Y (2017). Intercomparison of the Arctic sea ice cover in global ocean–sea ice reanalyses from the ORA-IP project. Clim Dynam.

